# Adult ADHD: Risk Factor for Dementia or Phenotypic Mimic?

**DOI:** 10.3389/fnagi.2017.00260

**Published:** 2017-08-03

**Authors:** Brandy L. Callahan, Daniel Bierstone, Donald T. Stuss, Sandra E. Black

**Affiliations:** ^1^Department of Psychology, University of Calgary Calgary, AB, Canada; ^2^Hotchkiss Brain Institute Calgary, AB, Canada; ^3^Sunnybrook Health Sciences Centre, Sunnybrook Research Institute Toronto, ON, Canada; ^4^LC Campbell Cognitive Neurology Research Unit, Sunnybrook Health Sciences Centre Toronto, ON, Canada; ^5^Faculty of Medicine, University of Toronto Toronto, ON, Canada; ^6^Hurvitz Brain Sciences Research Program, Sunnybrook Research Institute and University of Toronto Toronto, ON, Canada; ^7^Heart and Stroke Foundation Canadian Partnership in Stroke Recovery, Sunnybrook Health Sciences Centre Toronto, ON, Canada; ^8^Division of Neurology, Department of Medicine, Sunnybrook Health Sciences Centre and University of Toronto Toronto, ON, Canada

**Keywords:** ADHD, attention-deficit/hyperactivity disorder, dementia, MCI, diagnosis, elderly, adult

## Abstract

Attention-deficit hyperactivity disorder (ADHD) has historically been considered a disorder of childhood and adolescence. However, it is now recognized that ADHD symptoms persist into adulthood in up to 60% of individuals. Some of the cognitive symptoms that characterize ADHD (inability to provide sustained attention or mental effort, difficulty organizing or multi-tasking, forgetfulness) may closely resemble symptoms of prodromal dementia, also often referred to as mild cognitive impairment (MCI), particularly in patients over age 50. In addition to the overlap in cognitive symptoms, adults with ADHD and those with MCI may also share a number of behavioral and psychiatric symptoms, including sleep disturbances, depression, and anxiety. As a result, both syndromes may be difficult to distinguish clinically in older patients, particularly those who present to memory clinics with subjective cognitive complaints and fear the onset of a neurodegenerative process: is it ADHD, MCI, or both? Currently, it is unclear whether ADHD is associated with incipient dementia or is being misdiagnosed as MCI due to symptom overlap, as there exist data supporting either possibility. Here, we aim to elucidate this issue by outlining three hypothetical ways in which ADHD and MCI might relate to each other, providing an overview of the evidence relevant to each hypothesis, and delineating areas for future research. This is a question of considerable importance, with implications for improved diagnostic specificity of early dementia, improved accuracy of disease prevalence estimates, and better identification of individuals for targeted treatment.

## Introduction

Attention-deficit hyperactivity disorder (ADHD) is a neurodevelopmental disorder characterized by cognitive deficits (inability to sustain attention) and/or behavioral disturbances (inability to regulate motor behavior). The symptoms emerge before age 12 and disrupt participation in schoolwork, chores or interpersonal relationships (American Psychiatric Association, 2013). Originally considered exclusively a childhood disorder, it is now recognized that in 40–60% of cases, symptoms persist into adulthood and old age (Biederman, 2005; Adler et al., 2009; Culpepper and Mattingly, 2010; Volkow and Swanson, 2013), affecting ~2–4% of adults (Biederman, 2005; Kessler et al., 2006; Adler et al., 2009; Culpepper and Mattingly, 2010; Kieling and Rohde, 2012) and 3–4% of seniors (Michielsen et al., 2012; Kooij et al., 2016). Current criteria (American Psychiatric Association, 2013) include guidelines for diagnosing adults with ADHD; however, symptom onset must necessarily have occurred in childhood.

The presentation of ADHD has been described as changing from childhood to adulthood. Specifically, while overt behavioral disturbances tend to fade in adulthood (though not always; Brod et al., 2012; Guldberg-Kjär et al., 2013; Semeijn et al., 2015), cognitive symptoms (Volkow and Swanson, 2013; Asherson et al., 2016; Kooij et al., 2016) and subjective cognitive complaints (Bramham et al., 2012) seem to persist. The most common of these include difficulty engaging and sustaining attention, working memory deficits and slowed processing speed (Seidman, 2006). Often referred to generically as “executive deficits” (but see *Operational definitions*, below), these are thought to arise from compromised aspects of frontal lobe functioning. Fronto-striatal, frontal-temporo-parietal, and fronto-cerebellar networks have been documented as underdeveloped in ADHD compared to age-matched controls (Shaw et al., 2007; Cubillo et al., 2012). Memory problems have also been reported in adult ADHD, manifesting primarily as complaints of forgetfulness (Rosler et al., 2010). These symptoms can be similarly explained by impaired frontal-lobe functioning, as the frontal cortex is well-known to be involved in memory encoding and retrieval processes (Moscovitch and Winocur, 2002).

Although the above-described deficits are considered by some to be a core cognitive feature of ADHD (Willcutt et al., 2005; but see Castellanos et al., 2006; Bramham et al., 2012), they are certainly not unique to this disorder. Executive and memory deficits, broadly defined, have been described in many disorders, such as conduct disorder and autism (Sergeant et al., 2002). In adults and older adults, these types of impairments are often observed in the early stages of different types of dementias, including Alzheimer's disease (AD; Belleville et al., 2008; Saunders and Summers, 2010, 2011; Johns et al., 2012), dementia with Lewy bodies (DLB; Donaghy and McKeith, 2014), frontotemporal dementia (FTD; Rascovsky et al., 2011; Schubert et al., 2016), and vascular dementia (VaD; Vasquez and Zakzanis, 2015). For simplicity in the present review, we refer to the prodromal stage of all these dementias as mild cognitive impairment (MCI). MCI is conceptualized as an early expression of a neurodegenerative disorder, affecting cognition but not functional independence (Welsh-Bohmer, 2008; Benke, 2011), for which the pathological processes (e.g., proteopathy, tauopathy, synucleinopathy, etc.) are not yet known. The term “MCI” is currently often used to refer specifically to prodromal AD, particularly in its pure amnestic form. In the present review, we use the term as originally described by Petersen (2004), that is, to designate subjective complaints and performance below expected levels based on age and education in any cognitive domain, assumed to be due to any neurodegenerative process. Similarly, this closely relates to the definition of “mild neurocognitive disorder (mNCD)” as described in the Diagnostic and Statistical Manual of Mental Disorders, fifth edition (DSM-5) (American Psychiatric Association, 2013).

In addition to the substantial overlap in cognitive symptoms between ADHD and MCI in older adults, differential diagnosis is further complicated by the fact that these syndromes also share a number of clinically significant behavioral and psychiatric features. Namely, sleep disturbances are present in up to 70% of adults with ADHD (Asherson et al., 2016) and up to 59% of adults with MCI (Beaulieu-Bonneau and Hudon, 2009). Depression and anxiety, respectively, are observed in up to 44 and to 35% of adults with ADHD (Michielsen et al., 2013), and 27 and 14% of adults with MCI (Geda et al., 2008). All the above-mentioned disturbances are also well-known to have a deleterious impact on cognition (Salthouse, 2012; Yaffe et al., 2014).

The substantial overlap in cognitive and non-cognitive symptoms between adult ADHD and MCI can make both syndromes difficult to distinguish in older patients. Surveys of primary care physicians reveal that, although a majority of clinicians suspect some of their adult clients may have ADHD, most are not confident in their ability to accurately diagnose it (Adler et al., 2009). Although the prevalence may reach 4% in adults (Biederman, 2005; Kessler et al., 2006; Adler et al., 2009; Culpepper and Mattingly, 2010; Kooij et al., 2016), 40% of primary care physicians claim they have never encountered adult ADHD in their clinic, suggesting it goes unrecognized and undiagnosed (Fischer et al., 2012). In our own clinic, we have noted that accurate diagnosis is exceedingly difficult in adults with a lifelong history of cognitive difficulties who, as seniors, begin to complain of subjective memory problems and fear the onset of a neurodegenerative process: is it ADHD, MCI, or both? Other clinicians have raised the same concern (Fischer et al., 2012; Pollack, 2012). Ivanchak and Jicha (Ivanchak and Jicha, 2012) previously published an excellent comprehensive overview of the areas of overlap in genetics, neuroanatomy, and neurochemical pathways between ADHD and MCI and speculated whether ADHD may actually be associated with incipient dementia (i.e., be a form of MCI). Their review preceded the publication of several documents with key implications for the identification and differential diagnosis of ADHD in adults, including the DSM-5 (American Psychiatric Association, 2013), as well as articles on ADHD symptoms across the lifespan (Brod et al., 2012; Das et al., 2012, 2014; Kieling and Rohde, 2012; Michielsen et al., 2012, 2013; Guldberg-Kjär et al., 2013; Volkow and Swanson, 2013; McAuley et al., 2014; Semeijn et al., 2015; Asherson et al., 2016; Kooij et al., 2016), biological abnormalities and genetic risk factors for ADHD or inattention (Cubillo et al., 2012; Ducharme et al., 2012; Tomasi and Volkow, 2012; Sistino, 2013; Thapar et al., 2013; Faraone et al., 2014; Fried et al., 2014; Alemany et al., 2015, 2016; Dirlikov et al., 2015; Zhang et al., 2015), long-term outcomes of ADHD (Klein et al., 2012; Erskine et al., 2016; Hechtman et al., 2016; McAuley et al., 2017), data on the challenges in distinguishing ADHD from late-life cognitive impairment (Fischer et al., 2012), and physician-targeted guidelines to improve clinical diagnostic accuracy in the face of suspected ADHD or MCI (Bolea et al., 2012; Cortese and Castellanos, 2012; Pollack, 2012; Blackburn et al., 2014; Goodman et al., 2016).

Currently, the relationship between adult ADHD and MCI remains unclear. Updating and building upon Ivanchak and Jicha's previous report (Ivanchak and Jicha, 2012), the present review proposes formal, testable hypotheses and theoretical models, developed using existing published findings. We posit three hypotheses outlining potential ways in which ADHD and MCI might relate to each other: (1) ADHD and MCI represent two points along a single pathophysiological continuum; (2) ADHD increases the risk for MCI and dementia through an unrelated mediator; and (3) ADHD and MCI both manifest highly similar neurobehavioral symptoms through fundamentally distinct mechanisms (i.e., are unrelated). These hypothesized models are presented at a conceptually generic level, and do not attempt to integrate the myriad factors involved in the development of these highly multifactorial conditions. Moreover, the three hypotheses are not intended to be mutually exclusive; for instance, it is possible that ADHD may share common causal antecedent factors with MCI (Hypothesis 1) *in addition to* increasing risk for later MCI and dementia through an unrelated mediator (Hypothesis 2). Rather, these models are intended to complement each other by conceptually illustrating different aspects of the possible relationship between ADHD and MCI, which could guide future experiments aimed at untangling the possible links between the disorders. Lastly, as the specific etiologies of these highly heterogeneous conditions remain unknown, many of the elements presented within these frameworks are speculative and will need to be tested directly.

For clarity, we will begin by providing operational definitions of relevant terms used throughout this manuscript.

### Operational definitions

Throughout this paper, we use **ADHD** in reference to a formal diagnosis of ADHD according to DSM-5 criteria (American Psychiatric Association, 2013), which require: (A) a pattern of inattention and/or hyperactivity-impulsivity (≥6 inattentive and/or ≥6 hyperactive-impulsive symptoms) persisting for at least 6 months, that is inconsistent with developmental level; (B) symptom onset prior to age 12; (C) presence of symptoms in more than one setting (e.g., home, school, etc.); (D) disruption of social, academic, and/or occupational functioning due to symptoms; (E) that symptoms cannot be explained by another neuropsychiatric condition. In **adult ADHD**, only ≥5 inattentive and/or ≥5 hyperactive-impulsive symptoms are required, but symptom onset must still be prior to age 12 (American Psychiatric Association, 2013). We use **ADHD symptoms** to refer to isolated features of inattention and/or hyperactivity-impulsivity in adults that resemble features of ADHD, but that do not meet formal criteria (e.g., fewer than 5 inattentive and/or hyperactive-impulsive symptoms; symptom onset in adulthood or lack of compelling evidence for onset before age 12) and that could conceivably be secondary to other disease processes. ADHD symptoms are often measured in research studies using self- or informant-report questionnaires, such as the Conners' Adult ADHD Rating Scales (Conners et al., 1999) or the Adult ADHD Self-Report Scale (Kessler et al., 2005).

MCI and dementia are used to refer to different points along a single clinical course that is progressive, irreversible, and secondary to an underlying neurodegenerative process. **MCI** refers to an earlier point at which there are subjective cognitive complaints and cognitive deficits (typically defined as performance < −1–2 *SD* on at least one neuropsychological measure) but no functional impairment, whereas **dementia** refers to a later point at which there are cognitive *and* functional impairments (Petersen, 2004; American Psychiatric Association, 2013). In this paper, we also refer to specific forms of dementia, such as AD, FTD, DLB, or VaD, when the clinical symptoms fit a pattern that is characteristic of a particular pathological process. Although a considerable number of MCI cases do not go on to develop dementia, we assume that these represent misdiagnosed cases of non-degenerative neuropathological processes such as vasculopathy or hippocampal sclerosis (Britt et al., 2011).

In both the ADHD and MCI literature, the terms “attention,” “executive function,” and “frontal-lobe function” are often used interchangeably, despite considerable evidence that these are dissociable, non-unitary processes and the terms require definition (Stuss et al., 1995). **Frontal-lobe functions** refer to cognitive and emotional control processes than can be localized to selective anatomical regions within the frontal lobes (Stuss, 2011). They include the control of drive or activation, executive functions such as planning, switching and monitoring, emotional self-control, and metacognition. These are nodes of systems that interact with subcortical and posterior cortical regions. These control functions are less automated and fixed than posterior modular functions. As such, damage to many different brain regions may result in deficits in these control functions, particularly the cognitive control functions. The term **executive functions** is frequently used descriptively to identify these impairments, even though they involve neural networks that are not necessarily limited to the frontal lobes (Stuss et al., 1995; Stuss, 2011). These are typically assessed clinically using tests such as the Wisconsin Card Sorting Test (Heaton, 1981) and the Stroop Color-Word Task (Trenerry et al., 1989). **Attention**, in particular the Anterior Attentional System (or the Supervisory Attentional System) has been used to refer to a system of cognitive processes involving the activation and controlled maintenance of goal-directed thought. In this light, attention falls under the scope of executive functions. These processes are reflected in tasks such as sustained, selective, focused and divided attention (Stuss et al., 1995). A wide variety of tests are known to tap attentional processes, the Continuous Performance Test (CPT; Rosvold et al., 1956) being most commonly used in ADHD patients as it provides measures of multiple attentional processes at once.

Lastly, our use of the term **memory** reflects the encoding, storage, and retrieval of experiences associated with a specific temporospatial context (Moscovitch et al., 2016). We refer specifically to declarative memory processes, which involve interactions between several frontal-lobe and temporal-limbic regions (Moscovitch and Winocur, 2002). Recall and recognition of items within a word list or short story are typically the preferred measures for testing memory in both ADHD and MCI.

## Hypothesized relationships between ADHD and MCI

### ADHD and MCI represent two points along a single pathophysiological continuum

**Figure 1:**
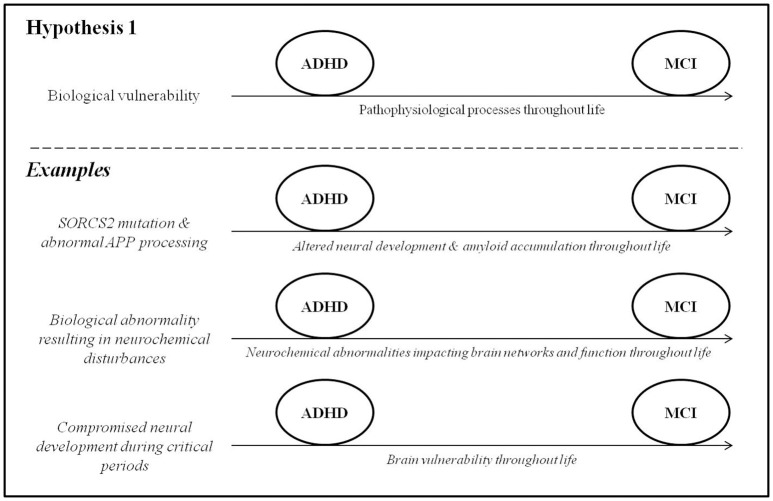
ADHD and MCI represent two points along a single pathophysiological continuum.

In this section, we draw on data from genetic and epidemiological studies to explore the possibility of a pathophysiological link between ADHD and MCI. In other words, ADHD may be caused by genetic and/or early life risk factors that impact brain development throughout life, manifesting clinically in childhood as hyperactivity/inattention and progressing to MCI and eventually potentially dementia in late adulthood (Figure 1). A key point in this model is that that some shared mechanism must precede the onset of difficulties in *both* ADHD and MCI.

#### Common genetic factors

Genetic variation is known to play a role in ADHD (Faraone et al., 2005) and in many dementias (e.g., Alemany et al., 2016; Erskine et al., 2016). It is important to state from the outset that genetic studies of these conditions have often yielded varied and inconsistent results, and a comprehensive review is beyond the scope of this paper. Here, we focus on one gene that has been implicated in both ADHD and AD and might shed light on a possible connection between them. Single-nucleotide polymorphisms in the gene *SORCS2* were found to associate with measures of inattention, assessed using the CPT, in a recent genome-wide association study of adult ADHD (Alemany et al., 2015). *SORCS2* is known to be involved in the processing of amyloid precursor protein (APP), which is a protein involved in neural growth and function (Zheng et al., 2006). One of the breakdown products of APP, amyloid-β, is a hallmark pathological feature of AD. Accordingly, *SORCS2* mutations have also been associated with increased risk for AD (Reitz et al., 2013). *SORCS2* might therefore constitute a potential shared genetic risk factor for ADHD symptoms and AD by altering both neurodevelopmental processes (in early life) and amyloid-β biology (into late adulthood). The possibility that changes in amyloid-β biology may constitute a pathophysiological link between ADHD symptoms and AD may be supported by several lines of behavioral evidence. First, decreased amyloid-β 42 levels in the cerebrospinal fluid, observed in preclinical AD (Fagan et al., 2007), have recently been associated with worse attentional control in cognitively normal healthy adults (Aschenbrenner et al., 2015) and may contribute to the cognitive symptoms that characterize ADHD (Alemany et al., 2015). Amyloid burden may also contribute to memory difficulties (Hedden et al., 2012), which are reported both in early AD (Belleville et al., 2008) and in ADHD (Rosler et al., 2010). At the least, the presence of ADHD early in life may reduce cognitive reserve to withstand amyloidosis later in life. This will require further investigation. Second, a knock-in drosophila model of AD, genetically engineered to over-express human amyloid-β precursor protein, unexpectedly exhibited behaviors strikingly similar to those of human ADHD, including hyperactivity that was responsive to dextroamphetamine (Zhang et al., 2015). Third, children with trisomy 21, who over-express APP and have overwhelmingly high rates of AD (Wiseman et al., 2015), also have significantly elevated rates (43.9%) of ADHD symptoms (Ekstein et al., 2011) compared to the rates of ADHD in the general population (up to 7.1%; Willcutt, 2012). It is worth adding a note of caution that the genetic contributions to ADHD have been difficult to identify due to large phenotypic and genetic heterogeneity, low penetrance, poor statistical power in many studies, and lack of definitive biomarkers identified so far (McAuley et al., 2014).

#### Common neurochemical disturbances

As reviewed by Prince (2008) and Cortese (2012), evidence from genetic and pharmacological studies has implicated the dysregulation of dopaminergic, noradrenergic, and serotoninergic circuitry as an important mediator of ADHD symptoms. Accordingly, findings from a large case-control study (Golimstok et al., 2011) suggest avenues for future exploration in this direction, specifically regarding a possible connection between ADHD and DLB. In this study, DSM-IV (American Psychiatric Association, 1994) diagnostic criteria for childhood ADHD were retrospectively applied to 109 older individuals with DLB, 251 with AD and 149 age-matched controls. When the prevalence of prior childhood ADHD was compared between the groups, it was found to be increased nearly threefold in the DLB patients (47.8%) compared to the AD and control groups (15.2 and 15.1%, respectively). The authors point out that the symptoms of ADHD and DLB are thought to stem from common neurobiological substrates, namely hypodopaminergic states in the brain, and speculate as to whether ADHD and DLB may therefore represent different points along a single pathophysiological continuum.

#### Common early-life risk factors

In addition to APP biology and neurochemical abnormalities, various early-life biological and social conditions are known to affect aspects of lifelong health and disease (Bateson et al., 2004) and may constitute shared risk factors for ADHD and MCI. The prenatal risk factors for ADHD have been well-characterized, the effects of which are largely mediated through impaired neurodevelopment or direct injury to the developing brain. These factors include fetal alcohol exposure, maternal smoking, low birth weight, prematurity, fetal distress, obstetrical complications, and congenital heart disease (Banerjee et al., 2007; Sistino, 2013; Thapar et al., 2013). Another crucial risk factor for ADHD is psychosocial adversity such as maltreatment, poverty, and social deprivation during critical periods of early brain development (Richards, 2013; Thapar et al., 2013). Some of these early-life factors are now being acknowledged as risk factors for MCI and dementia later in life. In particular, childhood maltreatment and low early-life socioeconomic status have been associated with evidence of compromised brain health (e.g., smaller hippocampi) that increase risk for dementia later in life (Whalley et al., 2006; Seifan et al., 2015). The exact pathophysiological mechanisms by which this occurs are unclear, partly owing to the decades-long gap between early-life risk factors and the development of MCI and dementia. In addition, several studies (summarized in Seifan et al., 2015) have shown that AD risk is increased in individuals with reduced body markers of early-life growth (height, limb length, and head circumference) that may be indicative of poor nutrition and, consequently, impaired brain development. These studies need to be interpreted cautiously, as many genetic, obstetrical, and developmental factors are known to affect growth and development.

#### Conflicting evidence

Aside from the findings reviewed above, there exists little evidence for a direct pathophysiological link between the ADHD and the eventual development of MCI or dementia. Most of the genetic polymorphisms that have been associated with ADHD (Faraone et al., 2014) do not seem to correspond to those found in the dementias (Loy et al., 2014). Certain important epidemiological characteristics of the disorders differ as well; for example, ADHD has a male predominance (Williamson and Johnston, 2015) despite the female-predominance of most dementias (Viña and Lloret, 2010).

Most importantly, a crucial corollary to this hypothesized model is that most, if not all, individuals with ADHD will eventually decline cognitively at a faster rate than otherwise healthy older adults later in life. At least some evidence indicates that this is not the case. In one study (Ivanchak et al., 2011), cognitive performance in multiple domains was compared between 13 older individuals with retrospectively-diagnosed childhood ADHD and 297 without. Twenty-three percent of cases with presumed childhood ADHD eventually developed MCI or dementia later in life, a statistically comparable proportion to individuals with no history of ADHD (21.5%). The authors conclude that ADHD is not associated with cognitive decline later in life, although this needs replication in independent samples as the ADHD group in this study was quite small. In addition, the presumed ADHD participants were not different on any of the neuropsychological measures in adulthood compared to the non-ADHD participants, suggesting that the presence of ADHD early in life has little or no bearing on the evolution of cognitive abilities in older age. In fact, one recent study found that ADHD symptoms in adults are more severe in middle-age than in old age (Das et al., 2014), further implying that ADHD is not associated with cognitive decline later in life.

### ADHD may increase risk for MCI through an unrelated mediator

**Figure 2:**
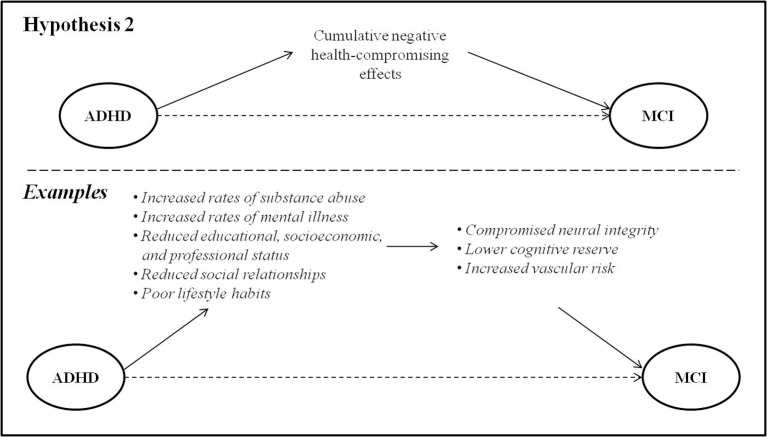
ADHD may be associated with risk factors in early and mid-life that lead to MCI via their deleterious effects on brain health.

Epidemiological studies have elucidated the role of certain lifestyle factors in increasing risk for the development of MCI and dementia. These include smoking, alcohol use, mental illness, diabetes and obesity, lower educational attainment and socioeconomic status, and lower quantity and quality of meaningful social relationships (Cohen et al., 2010; Boot et al., 2013). These risk factors are frequent in ADHD, and it is worth exploring the possibility that they may lead to MCI several decades later through their deleterious effects on vascular integrity, cognitive reserve, or overall brain health (Figure 2). A key point in this model, in contrast to the first, is that that the causative factor(s) must *follow* the onset ADHD but *precede* the onset of MCI.

#### Health-compromising behaviors

Individuals with ADHD have high rates of smoking and substance use disorders, compared to their age-matched peers (Biederman et al., 2006c; Klein et al., 2012; Volkow and Swanson, 2013; Erskine et al., 2016). Evidence suggests that these behaviors are primarily accounted for by inhibitory control deficits and difficulty regulating impulsivity (Ortal et al., 2015). There exist strong and consistent data linking smoking and heavy alcohol consumption to cognitive decline later in life, particularly with regard to executive functioning and memory (Lafortune et al., 2016). Several mechanisms are hypothesized to play a role in this association, the most well-recognized causal factor being compromised vascular health (Luchsinger et al., 2009; Baumgart et al., 2015). Diabetes and obesity, similarly frequent in ADHD, are also known to have negative impacts on vascular brain health and increase risk for dementia (Baumgart et al., 2015).

As it happens, behaviors that improve vascular health may represent a common *alleviating* factor in ADHD and dementia. Physical exercise is well-known to mitigate vascular risk, and has been shown to improve behavioral and neurocognitive symptoms in children with ADHD (Archer and Kostrzewa, 2012; Wigal et al., 2013; Cerrillo-Urbina et al., 2015) as well as improve cognitive function and decrease dementia risk in seniors (Baumgart et al., 2015).

#### Socioeconomic factors

Cognitive reserve is considered to be a measure of the brain's ability to withstand damage or neuropathology (Katzman et al., 1988) by recruiting a variety of brain networks and/or cognitive strategies developed through educational and occupational complexity (Cheng, 2016). Due to the cognitive and behavioral difficulties inherent to ADHD, educational and occupational attainment is typically limited in these individuals (Biederman and Faraone, 2006; Biederman et al., 2006a,c; Sobanski et al., 2007; Das et al., 2012; Klein et al., 2012; Hechtman et al., 2016). Indeed, early negative school experiences related to symptoms in childhood may result in a lack of interest and/or fundamental building blocks to pursue and succeed in postsecondary education. Consequently, opportunities for accumulating cognitive reserve may be limited. Lower vocational attainment also impacts later economic outcomes and results in worse financial stability for adults with ADHD (Biederman and Faraone, 2006; Hechtman et al., 2016), which has likewise been associated with increased risk of dementia later in life (Russ et al., 2013). In addition, lifelong lower socioeconomic status places individuals at increased risk for an unhealthy diet, potentially further exacerbating risk of dementia (Lafortune et al., 2016).

#### Psychosocial factors

There is also evidence that an active and socially-integrated lifestyle in late-life is protective against the development of dementia (Fratiglioni et al., 2004) implying that, conversely, social isolation may worsen the progression of dementia. Poorer social functioning, higher rates of divorce and greater social isolation have been identified among the adverse health and social outcomes of ADHD in adulthood and later life (Brod et al., 2012; Klein et al., 2012). These outcomes may constitute important links whereby ADHD in childhood or adulthood might increase risk for the development of MCI in later life.

Psychiatric comorbidities in ADHD may be another risk factor for later MCI or dementia. By young adulthood, individuals with ADHD show significantly increased vulnerability to anxiety and mood disorders compared to their age-matched peers (Biederman et al., 2006c). Lifetime psychiatric comorbidity in ADHD may reach 77%, with depression being the most common affective comorbidity (55%; Sobanski et al., 2007). The presence (Ownby et al., 2006), severity (Zilkens et al., 2014), and recurrence (Dotson et al., 2010) of depression in mid-life have been associated with risk for later dementia through inflammation, oxidative stress, and/or mitochondrial dysfunction that lead to neurodegenerative cell death (Kim et al., 2016), thus potentially mediating a relationship between ADHD and MCI.

The strong interactions between socioeconomic factors, psychological factors, and health-compromising behaviors must be acknowledged: these risk factors seldom present in isolation, and indeed some may be causally related to others (e.g., low socioeconomic status and poor diet). Their mediating effects on MCI in adults with ADHD are thus likely highly complex and multifactorial.

### ADHD and MCI manifest similar neurobehavioral symptoms through fundamentally distinct mechanisms

ADHD may possibly mimic the cognitive symptoms of MCI without being associated with incipient dementia. Experts in the field of dementia have urged emerging research to distinguish prodromal dementia syndromes from non-degenerative cognitive impairment in order to allow for the creation of appropriate treatment options (Blackburn et al., 2014). From a cognitive standpoint, ADHD and MCI manifest clinically similar symptoms which may be the cause of significant diagnostic confusion. In ADHD, the most consistently observed deficits are those relating to executive dysfunction, including deficits in initiation, response inhibition, impulse control, cognitive flexibility, and planning (Hervey et al., 1992). Impaired response inhibition has been proposed as a potential endophenotype for ADHD (McAuley et al., 2014), though it should be noted that this cognitive feature is also the most robust dysexecutive symptom in prodromal AD (Johns et al., 2012). The executive deficits in ADHD may additionally affect episodic memory downstream by impairing encoding and retrieval (Hervey et al., 1992; Moscovitch and Winocur, 2002). All of the above-described cognitive deficits can also be present in neurodegenerative syndromes: memory and executive functioning deficits may be observed in AD (Belleville et al., 2008; Saunders and Summers, 2010, 2011; Johns et al., 2012), DLB (Donaghy and McKeith, 2014), FTD (Schubert et al., 2016), and VaD (Vasquez and Zakzanis, 2015).

**Figure 3:**
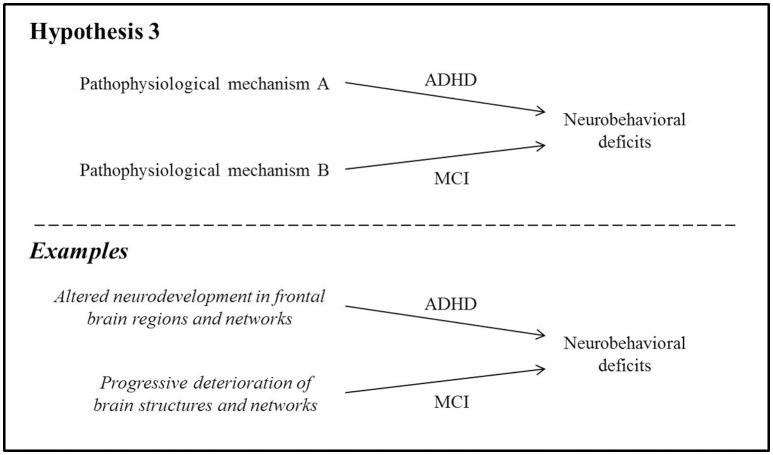
The pathophysiologies of ADHD and MCI are fundamentally unrelated despite similar neurobehavioral symptoms.

Here, we hypothesize that despite common neurobehavioral deficits, the pathophysiologies of ADHD and MCI are fundamentally unrelated, and that careful examination of clinical and neurostructural differences can help to disambiguate these syndromes (Figure 3).

#### Clinical differences

Some clinicians have maintained that adult ADHD and MCI can be adequately differentiated through thoughtful examination (Pollack, 2012), suggesting that their underlying etiologies are unique. The most crucial piece of evidence to support diagnosis is reliable retrospective report as to the onset and duration of cognitive concerns or changes: initial onset of neurobehavioral difficulties in adulthood is generally considered atypical in ADHD (although not unheard of Faraone et al., 2006b) and may be evidence of MCI (Pollack, 2012). In addition, patients with MCI typically report marked recent worsening in symptoms, despite being unable to provide a detailed history due to memory difficulties. In contrast, patients with ADHD may give a more detailed account of their difficulties, and these do not represent a decline from previous levels (Pollack, 2012). Moreover, medications that have typically been useful to some extent in relieving cognitive symptoms of dementia, including acetylcholinesterase blockers (donepezil, rivastigmine, galantamine) and N-methyl-D-aspartic acid antagonists (memantine), have had no clinically or statistically significant effects on cognition in adults with ADHD (Wilens et al., 2005; Biederman et al., 2006b), further suggesting that the underlying pathological etiologies of ADHD and dementia are distinct.

#### Neuroanatomical differences

Cognitive deficits in ADHD are believed to arise primarily from underdeveloped fronto-striatal, frontal-temporo-parietal, and fronto-cerebellar networks (Shaw et al., 2007; Cubillo et al., 2012) as well as changes in functional connectivity in the frontal lobes (Tomasi and Volkow, 2012). Structural atrophy in several brain regions is also present: in children, adolescents and young adults, studies have found reduced whole-brain volume (Wolosin et al., 2009; Batty et al., 2010) and cortical surface area (Wolosin et al., 2009; Dirlikov et al., 2015). Reduced gyrification of the cortex suggests delayed cortical maturation in children with ADHD (Wolosin et al., 2009), and frontal-lobe volumes have been found to be relatively smaller than those of age-matched controls, particularly medially (Ducharme et al., 2012). One study reported reduced cortical thickness specifically within the pars opercularis, thought to play a role in inhibitory control (Batty et al., 2010). These neuroanatomical differences are thought to contribute directly to various aspects of compromised frontal-lobe functioning. However, they do not typically deteriorate further over time in longitudinal follow-up of children and adolescents (Castellanos et al., 2002) and may actually improve following stimulant treatment (Cortese and Castellanos, 2012). It should be noted that these characteristics represent deviations from typical neuroanatomical findings in healthy individuals; despite this, persons with ADHD nonetheless often function at high social and occupational levels.

Some of these neuroanatomical findings are reminiscent of features of certain dementias. Most notably, reduced frontal-lobe volumes are characteristic of behavioral-variant FTD (bvFTD) (Whitwell et al., 2012; Valkanova and Ebmeier, 2014), and cortical thinning investigations have revealed significant thinning of the pars opercularis in both ADHD (Batty et al., 2010) and DLB (Blanc et al., 2015). Nearly all dementias also show some degree of whole-brain volume reduction that goes beyond expected age-related atrophy (Valkanova and Ebmeier, 2014). Despite some overlap between the neuroanatomical features of ADHD and those of MCI, the fact remains that none of the imaging findings in ADHD described thus far resemble the “signature” abnormalities observed in the dementias. These include, for instance, hippocampal atrophy and parietal hypometabolism in AD (Johnson et al., 2012; Risacher and Saykin, 2013), predominant frontal lobe atrophy with anterior temporal, parietal and cerebellar involvement in bvFTD (Whitwell et al., 2012), significant vascular disease in VaD (Román et al., 1993), and occipital hypoperfusion, focused atrophy of the midbrain, hypothalamus and subsantia innominata in DLB (Whitwell et al., 2007; Valkanova and Ebmeier, 2014).

Furthermore, it is assumed that cognitive impairment in MCI arises from progressive, irreversible deterioration of brain structures and/or networks involved in particular cognitive processes. For instance, cell death in entorhinal and hippocampal cortices (visible as atrophy on magnetic resonance imaging) is the hypothesized mechanism leading to episodic memory impairment in AD (El Haj et al., 2015; Tromp et al., 2015). Similarly, executive deficits in FTD have been attributed to significant neuronal loss and gliosis within the frontal lobes (Bang et al., 2015). These atrophic changes have been shown, at least in animal models, to be the direct result of the accumulation of neurotoxic proteins in the brain (e.g., tau pathology; Stancu et al., 2014).

Thus, neuroimaging evidence tends to support the hypothesis that neurobehavioral symptoms in ADHD and MCI manifest via distinct processes within the brain, namely *altered neurodevelopment* in the former and *progressive neurodegeneration* in the latter. That being said, neuroimaging investigations in ADHD subjects extending into mid- or late-life are sorely lacking, and this is a major gap that will need to be addressed in future explorations of the relationship between ADHD and MCI.

## Future directions

Several areas are ripe for exploration with regard to the relationship between ADHD and MCI. Specifically, we outline suggestions for future longitudinal, autopsy, neuroimaging, neuropsychological, and epidemiological studies that would be useful in addressing the issues raised in this review.

### Longitudinal studies

Long-term follow-up in adults with ADHD will ultimately provide the most compelling evidence to determine the extent to which ADHD is associated with progressive cognitive decline. Follow-up late into adulthood has been particularly challenging because of the relative newness of the ADHD diagnosis. Although descriptions of ADHD-like symptoms have existed for over 200 years (Lange et al., 2010; Bolea et al., 2012), the first formal diagnostic criteria for ADHD (then called “hyperkinetic reaction of childhood”) were only outlined in 1968 in the DSM-II (American Psychiatric Association, 1968). Even at that time, the diagnosis and treatment of hyperactive children remained poorly understood, and the number of formally diagnosed cases of ADHD only began to rise in the 1990s at which point it became recognized that many children did not simply outgrow their behavioral and attentional difficulties (Lange et al., 2010). As such, the largest cohorts of children with a formal diagnosis of ADHD are now nearing their thirties, and are not yet likely to be among those presenting to memory clinics with concerns of early dementia.

Nonetheless, certain studies have undertaken prospective longitudinal follow-up of children with ADHD into adolescence and adulthood (Castellanos et al., 2002; Biederman et al., 2006c; Gilliam et al., 2011; Klein et al., 2012; Hechtman et al., 2016). It will be necessary to follow these cohorts into their fifth and sixth decades of life in order to properly ascertain whether ADHD is a risk factor for dementia or simply a phenotypic mimic, taking care to assess multiple cognitive domains and to use alternate versions of neuropsychological instruments to avoid practice effects associated with repeated administration over time. Other questions that may be explored using longitudinal follow-up include determining whether individuals with ADHD who carry the *SORCS2* mutation are at increased risk for dementia compared to those without the mutation, and whether lower birth weight, prematurity, and obstetrical complications are common to both ADHD and late-life dementia. Alternatively, abnormal blinks and microsaccades, quantified using eye-tracking, have been suggested as potential indicators of ADHD (Fried et al., 2014) and might be assessed in older individuals to verify whether they associate with longitudinal measures of cognitive decline or dementia.

### Autopsy studies

Autopsy studies will be useful to outline the specific neuropathological changes that underlie the cognitive and behavioral symptoms of ADHD and to determine the extent to which these changes overlap with those of known dementias (e.g., plaques and tangles, Lewy bodies, vasculopathy). In the field of dementia, such studies have been instrumental in establishing gold standards for diagnosis (Durand-Martel et al., 2010) and in establishing clinico-pathological (Callahan et al., 2016) or imaging-pathological associations (Dallaire-Théroux et al., 2017) that can be used to guide or corroborate diagnoses *in vivo*. As such, the pathological substrates of the most common dementias have been relatively well-characterized. To our knowledge, no autopsy studies have been conducted in ADHD, though this is perhaps unsurprising due to the fact that current prospective cohorts of individuals with ADHD are relatively young and would be unlikely to come to autopsy. An example of such a study would involve assessing the presence of neuropathological features of major dementias (e.g., amyloid plaques, neurofibrillary tangles, Lewy body pathology, TDP-43, etc.) and comparing the frequencies of these pathologies between individuals with and without a history of ADHD. A certain amount of these pathologies can be expected within the context of normal aging (Snowdon, 2003), but higher levels in ADHD would suggest common pathophysiological processes with MCI and dementia. Unfortunately, autopsy studies remain a challenge as they must be performed on individuals who have died from causes or disease processes not affecting the brain.

### Neuroimaging studies

More realistic than autopsy studies, perhaps, would be to use *in vivo* methods to test these hypotheses, such as structural and functional neuroimaging. Such methods have been used in older adults to identify biomarkers of dementia (Valkanova and Ebmeier, 2014). While neuroimaging studies have been performed in younger participants with ADHD to document structural and functional abnormalities relative to age-matched peers (e.g., Castellanos et al., 2002), it will be crucial to undertake this research in older samples to determine whether specific dementia biomarkers are more prevalent in adults with ADHD. If so, this would provide support for the hypothesis that similar pathophysiological mechanisms are at play in both ADHD and MCI. Longitudinal neuroimaging work has thus far been limited to young participants, and direct comparisons of ADHD and MCI using imaging measures will be useful in elucidating this issue in adult samples. Furthermore, amyloid imaging in ADHD would help to determine whether pathologic protein aggregations in the brain are comparable to levels expected in AD.

### Neuropsychological studies

Neuropsychological studies will be important to establish cognitive differences between ADHD and MCI, particularly from a longitudinal perspective, and will provide answers as to whether ADHD is actually associated with increased risk for dementia. Relatively stable cognitive performance over time, interpreted in the context of normal age-related declines using reliable change indices (Gavett et al., 2015), will be a compelling argument in support of Hypothesis 3. If this is found to be the case, neuropsychological studies will be necessary to characterize the cognitive profiles specific to ADHD and MCI in older adults, so that clinicians can recognize the signs unique to each condition in the diagnostic process. Distinguishing between both syndromes clinically will be critical in order for affected individuals to receive disease-appropriate care and management. We propose that future work should aim, in particular, to clarify how ADHD and MCI differ in terms of specific executive functions. As discussed, both groups typically have some degree of impairment in executive functions as a whole, but it is unclear whether there exist group-specific deficits in particular components of executive functions (e.g., updating, initiation, interference, shifting). Identifying dysfunctional processes specific to ADHD and MCI will provide explicit ways in which these conditions may be distinguished using cognitive tests, and constitutes an initial step in the development of novel tests, if need be, that will be sensitive to these differences.

### Epidemiological studies

Epidemiological studies may provide answers to Hypothesis 2, regarding increased risk of MCI in ADHD via an unrelated mediator. Such studies might examine cohorts of adults with ADHD, with and without hypothesized mediators, in order to determine whether both groups differ in terms of later dementia risk. For example, a specific question of interest may be to determine whether older adults with symptoms of ADHD and a history of depression have higher rates of MCI than those with no history of depression. Work in this direction should also address the question of cognitive reserve, which to our knowledge has only been evaluated in children (McAuley et al., 2017), and remains a likely mediator of future decline. Adult cohorts of individuals with ADHD could be compared to other groups who manifest certain similar mediators of cognitive change (for example, adults with a history of childhood learning disabilities who may also have had limited opportunities to accrue cognitive reserve) to determine the specific contribution of the mediator to subsequent dementia risk. Here too, prospective longitudinal cohort studies would yield valuable insights.

## Conclusion

Adult ADHD shares many overlapping features with MCI, including cognitive deficits (particularly in memory and executive functioning) and psychiatric comorbidities (such as anxiety, depression, and sleep disorders). This has led to speculation that ADHD may be an incipient form of dementia, perhaps related to vascular or Lewy body pathology, through shared pathological mechanisms or via an unrelated mediator. Although there is a small number of intriguing findings that support a pathophysiological connection between ADHD and dementia, the bulk of the evidence presented in our review suggests that ADHD is a neurodevelopmental process fundamentally unrelated to MCI, and that any mechanistic link between the disorders is likely attributable to health-compromising mediators common in the ADHD population. Much of the diagnostic confusion that has been reported in memory clinics, including our own, presumably stems from phenotypical similarities between both syndromes. Of course, the possibilities outlined above are not mutually exclusive, and in some cases ADHD and MCI may overlap stochastically even without shared pathological pathways or mediators. Nevertheless, the hypotheses outlined above will need to be formally tested and replicated by independent research groups in order to fully ascertain the nature of relationship between these two conditions, and to determine whether the extent to which there exists phenotypical heterogeneity and possible subtypes in each syndrome.

Regardless of whether or not ADHD is a stage within a neurodegenerative process, current criteria for diagnosing MCI or dementia may not be appropriate or valid in individuals with a premorbid history of attentional and/or hyperactive difficulties. One core criterion for the diagnosis of a neurocognitive disorder is the need to establish “substantial impairment in cognitive performance, preferably documented using standardized neuropsychological testing” (American Psychiatric Association, 2013). However, standard cognitive measures as they are most commonly administered and interpreted in research studies on ADHD do not seem to capture the true executive difficulties of adults with ADHD, and results do not correlate well with self-reported functional outcomes (Barkley and Murphy, 2010; Asherson et al., 2016). Criteria for diagnosing neurocognitive disorders in adults with ADHD may therefore need to be validated and/or revised to rely more heavily on functional outcomes. Alternatively, future research should explore the development of new assessment tools to more accurately capture cognitive changes that may be reflective of a neurodegenerative condition in these individuals.

One major inescapable limitation of research in the area of adult ADHD is recall bias. Currently, a retrospective diagnosis of ADHD in adults relies on the presence of inattention and hyperactivity symptoms in childhood (i.e., before age 12; American Psychiatric Association, 2013). This implies that older individuals may have to think back 40 or 50 years when asked about these symptoms, and their responses may be biased by more recent events or by second-hand information. Retrospective biases may be all the more relevant when investigating potential associations with prodromal dementia, in which memory deficits are often a core feature and recall may be particularly unreliable. It is always advisable to gain collateral input from a knowledgeable informant when retrospectively assessing ADHD in older adults, if possible from someone who has known the participant throughout their lifetime (e.g., a sibling).

Nonetheless, informant reports may also be biased, and retrospective recall is not ideal for establishing diagnosis. At this time, it may be worthwhile for the medical community to consider the formulation of criteria for current ADHD symptoms specific to adults and seniors. In its most recent revision of the Diagnostic and Statistical Manual of Mental Disorders (DSM-5; American Psychiatric Association, 2013), the American Psychiatric Association increased the maximum age-at-onset criterion to age 12, based on a compilation of scientific evidence that the previous criterion of age 7 was too stringent and failed to account for many cases of ADHD (Faraone et al., 2006a,b). With increasing recognition that ADHD symptoms persist into adulthood and old age, and considering that the growing geriatric population will include individuals with ADHD, other authors have called for age-appropriate approaches to diagnosing ADHD later in life (Goodman, 2009; Bolea et al., 2012; Das et al., 2012, 2014; Goodman et al., 2016). Self-report rating scales or semi-structured clinical interviews are typically used to identify ADHD, however it is unclear whether these methods are appropriate for older samples. Of the publicly available self-report scales (Ward et al., 1993; Conners et al., 1999; Kessler et al., 2005; Barkley, 2011) or clinical interviews (DuPaul et al., 1998; Barkley and Murphy, 2008; Kooij, 2012; Marchant et al., 2013) for the assessment of ADHD symptoms in adults >18 years, only one has been validated for use in adults >60 years to our knowledge (Semeijn et al., 2013), and even it was validated against a tool that has not yet itself been validated (Kooij, 2012). At least one empirical study suggests that certain screening tools and rating scales may be very sensitive, but not specific, for detecting ADHD in adults (Hines et al., 2012). Thus, it is possible that existing tools may inaccurately capture the features of ADHD in older samples, and it will be necessary to determine the psychometric properties of screening tools used to quantify symptoms in these individuals. It may be time to consider the development of adult-specific criteria or instruments that do not rely on retrospective report and that are well-validated for use in older cohorts.

The present paper extends previous work beyond a descriptive analysis of the overlapping features of both syndromes, and provides a framework that can guide the design of research studies in this area. Further work should include syndromatic as well as symptomatic cases of ADHD, prospective longitudinal cohort studies and, eventually, autopsy studies in order to broaden our current knowledge of this under-studied area of adult neuropsychology. This is crucial for several reasons. First, improved accuracy of disease incidence and prevalence rate estimates will have important socio-economic impacts, both direct and indirect (Bolea et al., 2012). Second, teasing apart individuals who are in the early stages of dementia from those who are symptomatically similar but non-degenerative will allow for more appropriate recruitment into clinical trials and research studies specifically focused on dementia. Third, determining whether ADHD is truly associated with increased risk of cognitive decline is critical to provide direction for the assessment and management of these patients, and is the first step toward the study and development of pharmacologic and behavioral avenues for intervention. Continued research in this area will inform the need for, and formulation of, diagnostic criteria for MCI and dementia adapted to seniors with ADHD, as well as for ADHD in adults and seniors.

## Author contributions

BC and DB conceived the question of interest, conceptualized and developed the models, performed the literature review and wrote the manuscript. DS and SB made substantial contributions to the conception and design of the models, and revised the manuscript critically for important intellectual content. All authors commented on the manuscript at all stages and consent to their names appearing on it.

### Conflict of interest statement

The authors declare that the research was conducted in the absence of any commercial or financial relationships that could be construed as a potential conflict of interest.
